# Association of Hyperuricemia with Impaired Left Ventricular Systolic Function in Patients with Atrial Fibrillation and Preserved Kidney Function: Analysis of the POL-AF Registry Cohort

**DOI:** 10.3390/ijerph19127288

**Published:** 2022-06-14

**Authors:** Marcin Wełnicki, Iwona Gorczyca-Głowacka, Arkadiusz Lubas, Wiktor Wójcik, Olga Jelonek, Małgorzata Maciorowska, Beata Uziębło-Życzkowska, Maciej Wójcik, Robert Błaszczyk, Renata Rajtar-Salwa, Tomasz Tokarek, Jacek Bil, Michał Wojewódzki, Anna Szpotowicz, Małgorzata Krzciuk, Monika Gawałko, Agnieszka Kapłon-Cieślicka, Anna Tomaszuk-Kazberuk, Anna Szyszkowska, Janusz Bednarski, Elwira Bakuła-Ostalska, Beata Wożakowska-Kapłon, Artur Mamcarz

**Affiliations:** 1 3rd Department of Internal Medicine and Cardiology, Medical University of Warsaw, 02-091 Warsaw, Poland; marcin.welnicki@wum.edu.pl (M.W.); wpwojcik@gmail.com (W.W.); artur.mamcarz@wum.edu.pl (A.M.); 2 1st Clinic of Cardiology and Electrotherapy, Swietokrzyskie Cardiology Centre, Jan Kochanowski University, Collegium Medicum, 25-369 Kielce, Poland; olga_jelonek@wp.pl (O.J.); bw.kaplon@poczta.onet.pl (B.W.-K.); 3 Department of Internal Medicine, Nephrology and Dialysis, Military Institute of Medicine, 04-141 Warsaw, Poland; alubas@wim.mil.pl; 4 Department of Cardiology and Internal Diseases, Military Institute of Medicine, 04-141 Warsaw, Poland; mmaciorowska@wim.mil.pl (M.M.); buzieblo-zyczkowska@wim.mil.pl (B.U.-Ż.); 5 Department of Cardiology, Medical University of Lublin, 20-059 Lublin, Poland; m.wojcik@umlub.pl (M.W.); robertblaszczyk1@wp.pl (R.B.); 6 Department of Cardiology and Cardiovascular Interventions, University Hospital, 30-688 Krakow, Poland; rajfura@op.pl (R.R.-S.); tomek.tokarek@gmail.com (T.T.); 7 Department of Invasive Cardiology, Centre of Postgraduate Medical Education, Central Clinical Hospital of the Ministry of Interior and Administration, 02-507 Warsaw, Poland; biljacek@gmail.com (J.B.); michaljerzywojewodzki@gmail.com (M.W.); 8 Department of Cardiology, Regional Hospital, 27-400 Ostrowiec Swietokrzyski, Poland; szpotowiczanna@wp.pl (A.S.); mekrzciuk@gmail.com (M.K.); 9 1st Chair and Department of Cardiology, Medical University of Warsaw, 02-097 Warsaw, Poland; mgawalko@wum.edu.pl (M.G.); agnieszka.kaplon@gmail.com (A.K.-C.); 10 Department of Cardiology, Maastricht University, Universiteitssingel 50, 6229 ER Maastricht, The Netherlands; 11 Institute of Pharmacology, West German Heart and Vascular Centre, University Duisburg-Essen, Forsthausweg 2, 47057 Duisburg, Germany; 12 Department of Cardiology, Medical University, 15-276 Bialystok, Poland; a.tomaszuk@poczta.fm (A.T.-K.); annaszyszkowska92@gmail.com (A.S.); 13 Department of Cardiology, St John Paul II Western Hospital, 05-825 Grodzisk Mazowiecki, Poland; medbed@wp.pl (J.B.); elwira.bakula@gmail.com (E.B.-O.)

**Keywords:** atrial fibrillation, hyperuricemia, diuretics, renal function, left ventricular ejection fraction

## Abstract

Hyperuricemia is associated with the risk of developing atrial fibrillation (AF) and heart failure. However, coexisting chronic kidney disease and certain cardiovascular drugs make it difficult to determine whether hyperuricemia is a risk factor or merely a marker of pathology. We retrieved data from the Polish Atrial Fibrillation (POL-AF) registry, which included consecutive patients hospitalized with AF from January to December, 2019. We included 829 patients (mean age: 72.7 ± 11.1 years) with data on serum uric acid (UA, mean: 6.56 ± 1.78 mg/dL) and estimated glomerular filtration rate (eGFR) ≥ 60 mL/min/1.73 m^2^. We found that UA and ejection fraction (EF) were significantly correlated (r = −0.15, *p* < 0.05), but not EF and eGFR or eGFR and UA. A multiple regression analysis adjusted for age, body mass index, eGFR, and UA, showed that UA was significantly associated with a reduced EF (R^2^: 0.021; *p* < 0.001). The UA cut-off indicative of an EF < 40% was 6.69 mg/dL (AUC, area under the curve: 0.607; 95% CI: 0.554–0.660; *p* = 0.001). Among drugs known to effect UA concentrations, we found that only diuretics were used more frequently in patients with high UA (above the median) than in patients with low UA (77.5% vs. 67%, *p* < 0.001). Among patients that used diuretics, UA remained significantly correlated with EF. Thus, we showed that reduced EF was associated with UA in patients with AF and normal renal function, independent of eGFR and diuretic use.

## 1. Introduction

There is no doubt that atrial fibrillation (AF) and heart failure (HF) are two of the most serious challenges in cardiology in the 21st century [1,2]. AF is the most common supraventricular tachyarrhythmia. It affects 2–4% of the general population, and it is associated with a significant increase in the risk of cardiovascular incidents [1]. The risk of death in patients with AF is 1.5–3.5 fold higher than in the general population. The main causes of increased mortality include stroke and HF [1]. Indeed, 20–30% of all ischemic strokes occur in patients with AF. Moreover, it is estimated that left ventricular dysfunction and/or clinically overt HF occur in 20–30% of patients with AF [1]. AF can be either a cause or a consequence of HF [1,2]. In recent years, cardiologists have been increasingly interested in the problem of asymptomatic hyperuricemia. Although in the general population, the upper limits of normal for serum uric acid (UA) are 7 mg/dL (420 µmol/L) for men and 6 mg/dL (360 µmol/L) for women, among patients at high cardiovascular risk, the suggested goal is a UA concentration below 5 mg/dL (297 µmol/L) [3,4,5]. The studies conducted to date have shown that hyperuricemia is a risk factor for metabolic syndrome, carbohydrate disorders, hypertension, stroke, and cardiovascular events, including fatalities [6,7,8,9,10,11,12]. It has also been shown that hyperuricemia increases the risk of developing AF and worsens the prognosis of patients with HF [13,14,15,16,17]. However, data are scarce on the role of hyperuricemia in patients with existing AF. In a previous analysis, we showed that UA levels above 6.9 mg/dL comprised a marker of reduced EF (EF < 40%) in patients with AF [18]. However, considering the relationship between hyperuricemia and renal function and the correlation between EF and renal function, it is difficult to determine unequivocally whether hyperuricemia is an independent risk factor that precedes the occurrence of myocardial damage or whether it is an indicator that damage has occurred. Chronic kidney disease (CKD) and HF frequently coexist. According to a meta-analysis by Damman et al., CKD was found in approximately half of patients with HF. Other studies have indicated that kidney damage occurred in 43% of patients with chronic HF and in 53% of patients with acute HF [19,20,21]. Many studies have also shown that AF increased the risk of CKD, and vice versa [22,23]. Moreover, the pathomechanism of the relationship between hyperuricemia and HF remains unclear. One of the postulated common denominators is renal impairment. Another potential mechanism, however, is increased xanthine oxidase (XO) activity. The effects of XO include both an increase in the UA concentration and an increase in the production of free oxygen radicals [16,17]. Increased oxidative stress promotes cardiomyocyte hypertrophy, cardiac remodeling, and impaired myocardial contractility. Some authors suggest that hyperuricemia in the course of HF is only a marker of increased oxidative stress, due excessive XO activity [17]. In patients with AF, arrhythmia dynamics often change over time, which contributes to the variable severity of HF symptoms. Therefore, we reasoned that this population could be ideal for investigating the relationship between hyperuricemia and HF. Moreover, it is important to consider the effects of drugs commonly used in cardiology that can affect uric acid levels [4,5]. Bearing these factors in mind, we decided to conduct an analysis of the data in the POL-AF registry, but only for patients with available data on serum UA levels and with estimated glomerular filtration rates (eGFR) ≥ 60 mL/min/1.73 m^2^. Our primary aim was to investigate whether the relationship between UA and EF in patients with AF might be independent of renal function. Our secondary aim was to assess the effects of selected drugs on that relationship.

## 2. Materials and Methods

### 2.1. Study Design and Participants

The Polish Atrial Fibrillation (POL-AF) Registry study was a multicenter, prospective, observational study that included patients with AF from 10 cardiology centers. The study was registered in ClinicalTrials.gov (study ID: NCT04419012). The data were collected for 2 full weeks each month, from January to December 2019. Eligible patients were over 18 years old, were diagnosed with AF, documented with an electrocardiographic examination or medical report, and were hospitalized for either urgent or planned reasons. No clear exclusion criteria were defined; however, patients admitted for an ablation due to AF were excluded. The study was approved by the Ethics Committee of the Swietokrzyska Medical Chamber in Kielce (104/2018). The Ethics Committee waived the requirement for informed consent from the patients.

**Figure 1:**
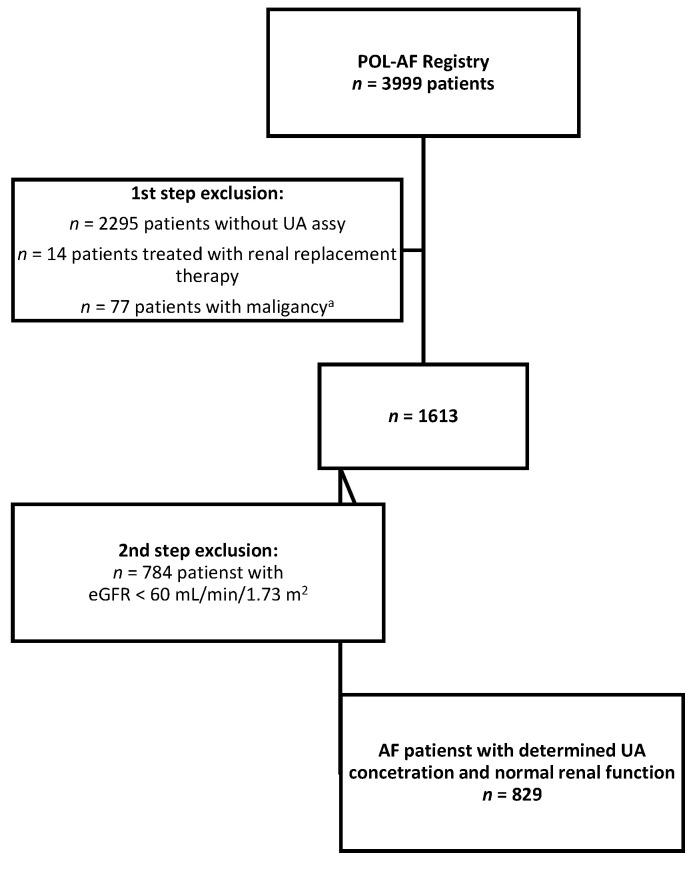
Flowchart shows the study selection process. Abbreviations: UA, uric acid; eGFR, estimated glomerular filtration rate. ^a^ A malignancy was defined as active cancer or a cancer treatment completed less than one year before the date of registration.

The present study was an extension of the POL-AF study, which was conducted as a registry study, and it did not affect the standard diagnostic and therapeutic procedures carried out in the individual centers. The POL-AF registry did not include prospective observations, and no biological material was collected or stored as part of the registry. The fact of including the patient in the registry did not affect the selection of the tests performed. We aimed to analyze the importance of UA in the study population. From the original group of 3999 patients included in the POL-AF registry, the UA concentration was determined in 1704 patients. From those, we excluded patients treated with renal replacement therapy (*n* = 14), with malignant diseases (*n* = 77), and with eGFR < 60 mL/min/1.73 m^2^ (*n* = 784, eGFR was calculated for each patient). Thus, 829 patients were included in the final analysis, including 366 women (44% of the studied population; Figure 1).

Data were collected on baseline characteristics, including demographics, medical history, diagnostic test results, and pharmacotherapy. Impaired kidney function was defined as eGFR < 60 mL/min/1.73 m^2^ (according to the short MDRD, Modification of Diet in Renal Disease, formula). We also collected data on pharmacotherapy regimens, including the use of beta-blockers (BB), angiotensin converting enzyme inhibitors (ACEi), angiotensin receptor blockers (ARB, sartans), and diuretics (including aldosterone receptor antagonists [ARA], loop diuretics, and other diuretics). The data from the registry indicated the use of a given group of drugs, but lacked information on doses.

### 2.2. Patient Grouping

Previous studies demonstrated a relationship between UA and EF <40% in patients with AF. However, pharmacotherapy regimens that are standard for HF with reduced EF (HFrEF), particularly diuretics, can affect the concentration of UA. Because the UA cut-off point identifies patients with EF < 40%, we could expect a significant difference in EF between patients treated and those not treated with standard HF pharmacotherapy regimens, particularly diuretics. However, if patients were divided according to the objective UA value into two equal groups (according to the median UA value), the influences of UA on the EF and the frequency of pharmacotherapy regiments would no longer be clear. In the present our study, we aimed to evaluate the relationship between UA and EF, independent, as much as possible, from the potential impact of pharmacotherapy. To that end, we used the median concentration of UA as the criterion for dividing the patients into groups, instead of a UA cut-off point for EF < 40%.

### 2.3. Statistical Analysis

Continuous data are expressed as the means and standard deviations and categorical data are expressed as frequencies and percentages. We performed parametric tests to analyze continuous variables with a normal distribution; and we performed non-parametric tests for variables with non-normal distributions. Group comparisons were performed with the Student’s *t*-test or the Mann–Whitney U test, for continuous variables, and the chi-squared test for categorical variables. We performed Pearson’s correlation analyses to investigate relationships between variables. For logistic regression models, we report the odds ratios (OR) with 95% confidence intervals (95% CI). We built a multiple regression model with backward stepwise calculations. The model included age, body mass index (BMI), eGFR, and UA concentration. In the logistic regression model, we took into account the fact of using particular groups of drugs and the diagnosis of permanent AF. To determine the predictive value of variables, we performed receiver-operating characteristic (ROC) curves, and set cut-off points according to the Youden index. Two-tailed *p*-values < 0.05 were considered significant. Missing data were removed case-wise. All statistical analyses were performed with Statistica 13 (TIBCO Software Inc., Palo Alto, CA, USA).

## 3. Results

### 3.1. General Characteristics

The cohort had a mean age of 72.7 ± 11.1 years, 366 were women (44% of the study population), and the mean UA was 6.56 ± 1.78 mg/dL. The general norms of UA are different for men and women; however, in our study population, there was no significant difference in the mean UA concentrations between women (6.520 mg/dL) and men (6.613 mg/dL; *p* = 0.0436). The mean EF was 49.9 ± 12.5%. Among the comorbidities, arterial hypertension was the most common (86% of patients), and EF < 40% was found in 16% of the cohort. The general characteristics of the study population are presented in Table 1.

### 3.2. Uric Acid

**Figure 2:**
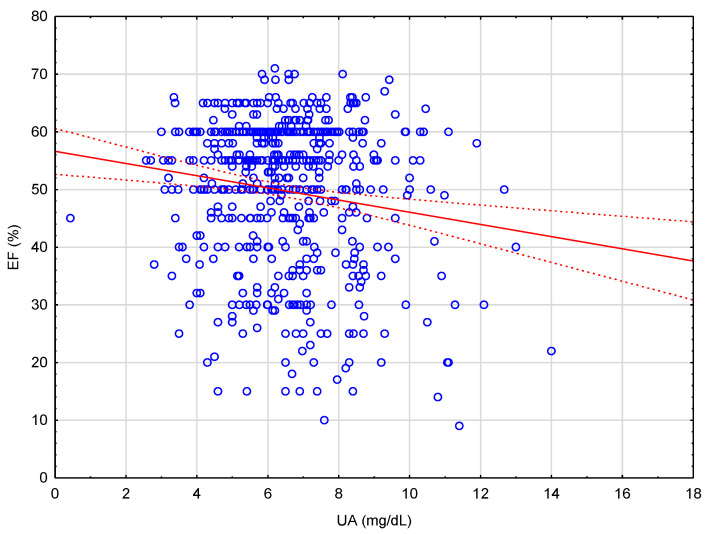
Scatterplot shows EF (%) as a function of UA (mg/dL). Cases with missing data were excluded; linear regression fit: EF (%) = 56.613 − 1.056 * UA (mg/dL), r = −0.1458.

A correlation analysis showed that the UA concentration was inversely correlated with the left ventricular EF. However, the UA concentration was not correlated with eGFR (Table 2 and Figure 2).

In the multiple backward stepwise regression analysis model, we included age, BMI, eGFR, and UA (Table 3); we found that only UA was independently associated with EF (R^2^ = 0.021; *p* < 0.001) in our study population.

**Figure 3:**
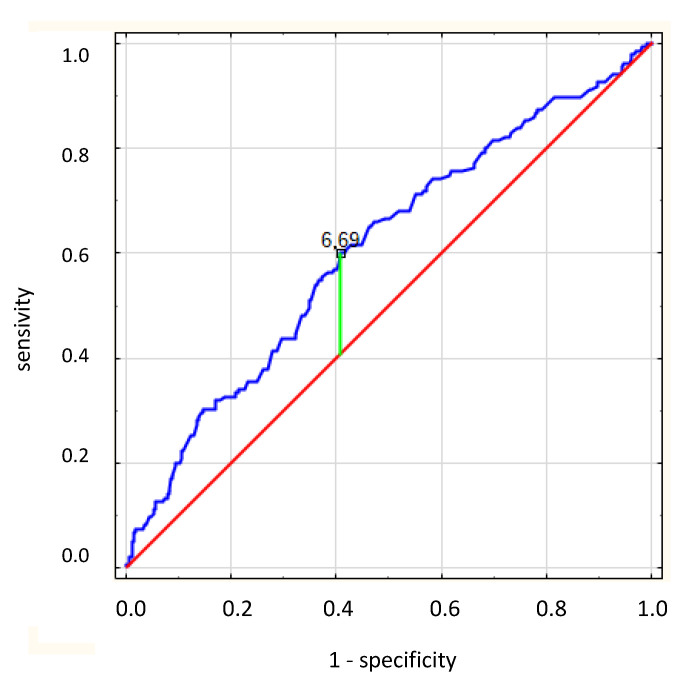
A receiver operating characteristic curve shows the prognostic value of UA concentration for predicting which patients will have an ejection fraction < 40%. The cutoff value (6.69 mg/dL) was identified with the Youden index method. AUC: area under the curve.

Based on the ROC curve (Figure 3), we found that the UA concentration of 6.69 mg/dL was the best cut-off point for predicting a reduced EF (<40%) in patients with AF. The area under the curve was 0.607 (95% CI: 0.554–0.66, *p* = 0.001), and the predictive ability showed approximately 60% sensitivity and 60% specificity. According to the univariate logistic regression analysis, patients with a UA ≥ 6.69 mg/dL were about twice as likely to have an EF < 40% than those with a UA < 6.69 mg/dL (OR: 2.17, 95% CI: 1.48–3.15; *p* < 0.001).

### 3.3. Comparison of Patients with High vs. Low Uric Acid Concentrations

**Figure 4:**
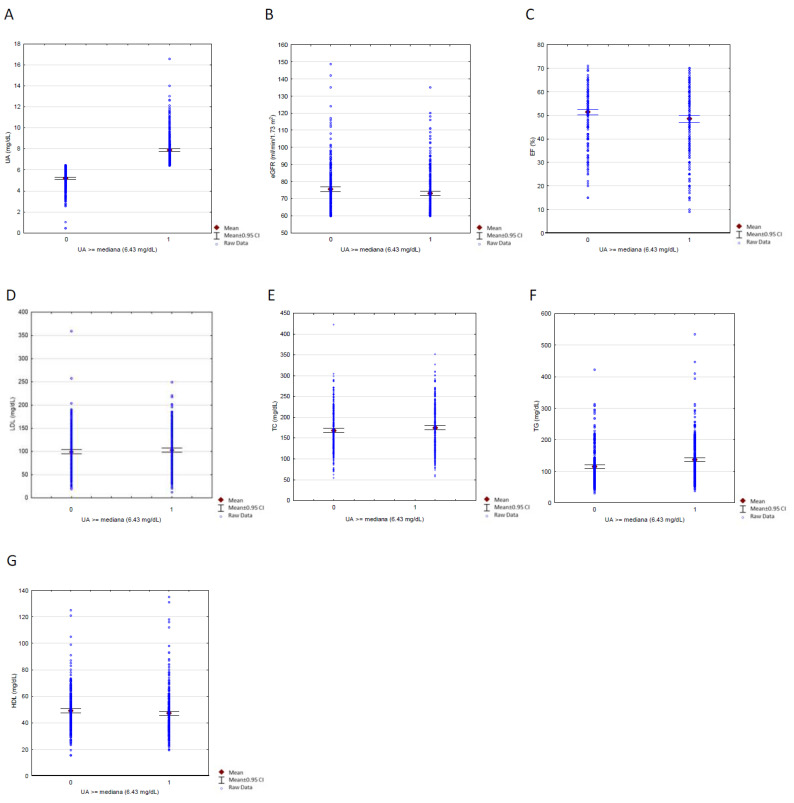
Scatterplots with error bars for comparison of groups, based on median UA concentration (6.43 mg/dL), as far as fallowing variables are concerned: (**A**) eGFR, (**B**) UA concentration, (**C**) EF, (**D**) LDL concentration, (**E**) TC concentration, (**F**) TG concentration, (**G**) HDL concentration. Abbreviations: UA, uric acid; EF, ejection fraction; eGFR, estimated glomerular filtration rate; TC, total cholesterol; LDL, low-density lipoproteins; TG, triglycerides; HDL, high-density lipoproteins; CI, confidence interval.

**Figure 5:**
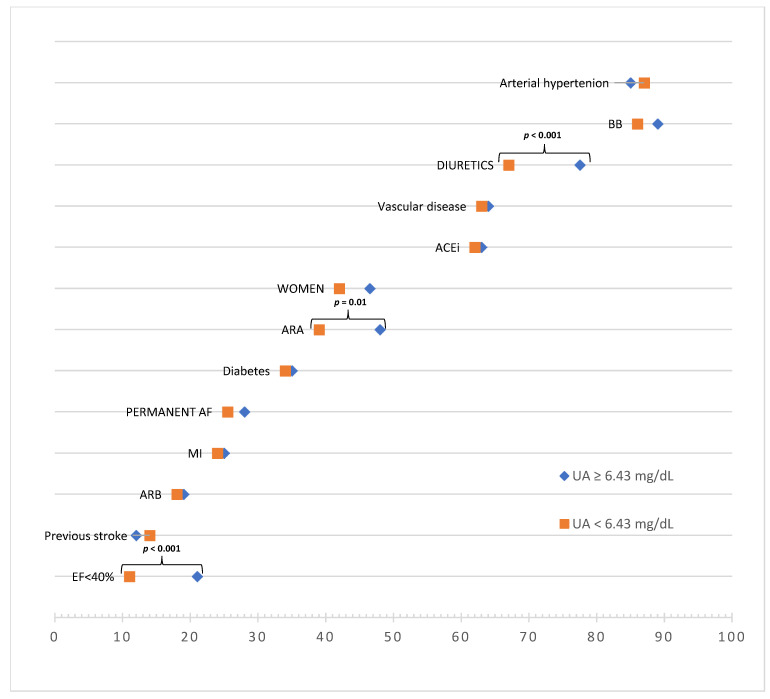
Comparison of groups, based on median UA concentration (6.43 mg/dL), as far as EF, diuretics usage and type of AF are concerned. Abbreviations: UA, uric acid; atrial fibrillation; EF, ejection fraction; ARA, aldosterone receptor antagonists; diuretics stands for: any and all diuretics, including ARA or/and loop diuretics.

To minimize the impact of the standard HFrEF pharmacotherapy regimen on our results, as described in Section 2.2, we divided patients into two subgroups of high and low UA concentrations, based on the median concentration of the study population (6.43 mg/dL). The results of this comparison are shown in Table 4 and Figure 4 and Figure 5.

Patients with high UA concentrations were distinguished by a lower mean EF and a higher frequency of EF < 40% compared to those with low UA concentrations. In the context of pharmacotherapy, these groups only differed significantly in the use of diuretics (i.e., ARA, specifically, and diuretics, in general).

**Figure 6:**
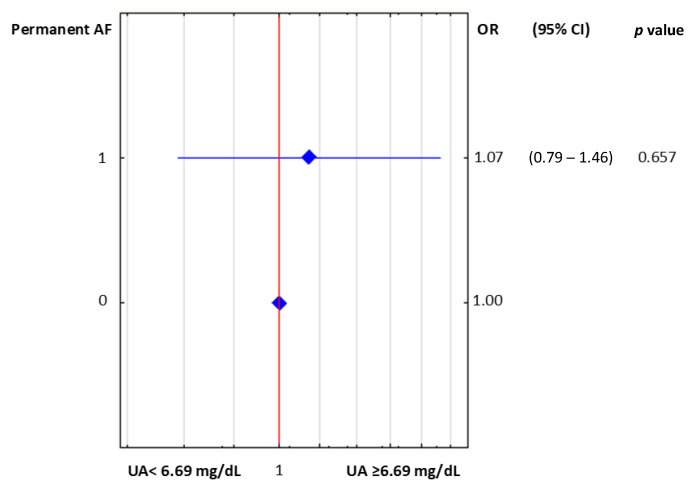
Results of univariate logistic regression for permanent AF and UA cutoff value (6.69 mg/dL). Abbreviations: AF, atrial fibrillation; UA, uric acid; OR, odds ratio; CI, confidence interval.

The difference in mean eGFR values requires an additional comment. This difference resulted from adopting the median UA concentration as the criterion for dividing the studied population. In an additional analysis, when the division criterion was UA = 6.69 mg/dL (according to the ROC for EF < 40%), the mean eGFR values did not differ significantly between groups (75 mL/min/1.73 m^2^ vs. 73 mL/min/1.73 m^2^, respectively, for UA < 6.69 mg/dL and for UA ≥ 6.69 mg/dL; *p* = 0.071). It is worth noting that, with that division criterion, we found significant differences in the mean concentrations of HDL and TG (i.e., significantly lower and higher, respectively, in the UA ≥ 6.69 mg/dL group, compared to the UA < 6.69 mg/dL group). It should also be noted that the type of AF did not have a significant effect on whether the UA was above or below 6.69 mg/dL. In a univariate logistic regression analysis, permanent AF was not associated with UA concentration ≥ 6.69 mg/dL (OR: 1.07, CI: 0.79–1.46; *p* = 0.657). The results of this univariate logistic regression are presented in Figure 6.

### 3.4. Drug Effects

**Figure 7:**
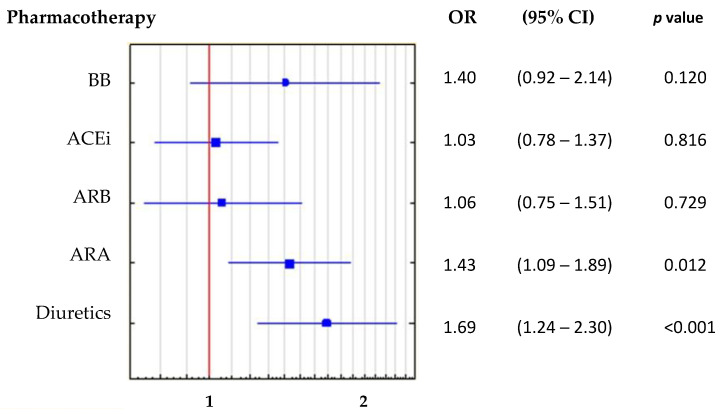
Results of univariate logistic regression for all groups of drugs. Abbreviations: BB, beta-blockers; ACEi, angiotensin converting enzyme inhibitors; ARB, angiotensin receptor blockers; ARA, aldosterone receptor antagonists; OR, odds ratio; CI, confidence interval. Diuretics: any and all diuretics, including ARA or/and loop diuretics.

We performed a logistic regression model to analyze all groups of drugs used by the included patients. However, only the use of diuretics was significantly associated with a UA above the median. The probability of having a UA concentration equal to or above the median value increased by 43% with the use of ARA (OR: 1.43, 95% CI: 1.09–1.88, *p* = 0.012), and increased by 69%, with the use of any diuretic (OR 1.69, 95% CI: 1.24–2.30, *p* < 0.001). The results of univariate logistic regression are presented in Figure 7. However, the backward stepwise regression model results indicated that only the use of diuretics, in general, remained significantly associated with a high UA.

After obtaining these results, we performed the correlation analysis again, but this time, we only included patients that used diuretics (in general). The results of this analysis are presented in Table 5. We observed a persistent significant correlation between UA and EF, and again, no significant correlation between UA and eGFR.

## 4. Discussion

In the prospective Chronic Renal Insufficiency Cohort study, the prevalence of AF was 18% in patients with CKD [24]. Moreover, about half of the patients with AF had impaired renal function [25,26]. Our population was consistent with those data; out of our original group of 1613 patients with AF, 829 had preserved kidney function [18]. We have shown that in patients with AF and eGFR > 60 mL/min/1.73 m^2^, hyperuricemia is a marker of reduced EF and the relationship between UA and EF is independent both of renal function and the use of drugs typical for treating heart failure. These three issues need to be discussed.

### 4.1. Heart Failure, Renal Failure, and Hyperuricemia—A Toxic Triangle

It is known that the concentration of UA increases with deteriorating kidney function. On the other hand, an increasing number of studies have shown that, in patients with normal kidney function, high UA levels may lead to kidney damage [27,28]. Moreover, renal function is often affected in patients with HFrEF [29]. Thus, the interplay between heart and kidney function is complex, and this complexity gives rise to the multiplicity of cardio-renal syndromes [30]. Consequently, it remains difficult to state unequivocally whether hyperuricemia is the cause or the result of deteriorating renal function and/or progressive left ventricular systolic dysfunction, among patients with AF and reduced EF. The complexity of this relationship, in terms of pathophysiology, was described by Kumric et al. [31], among others. In the course of progressive HF, XO activity can be stimulated by hypoxia, catabolism, and cell apoptosis. Elevated XO activity then leads to oxidative stress. The coexistence of tissue hypoperfusion leads to the simultaneous overproduction of UA and a complex cardiotoxic effect [31]. At the same time, UA excretion in the urine is impaired, due to an increased concentration of lactic acid and to hypoperfusion of the kidneys [30,31]. It is also believed that the drugs commonly used in HF may play important roles. Therefore, our results are important, in the context of interpreting the interrelationships between UA and EF in patients with AF.

### 4.2. Uric Acid Separate from Kidney Function

To our knowledge, our observations are the first of this type described in an AF patient population. Moreover, our results were consistent with observations from other studies regarding the importance of hyperuricemia in assessing the prognosis of patients with HFpEF. Palazzuoli et al. showed that hyperuricemia, but not CKD, was an independent predictor of hospitalization due to HF or death in a HFpEF subpopulation (HR: 2.38, 95% CI: 1.32 to 4.28; *p* = 0.004) in a multivariate analysis [32]. That prospective study had a follow-up of 6 months, and hyperuricemia was defined as UA ≥ 7.0 mg/dL in men and ≥ 6 mg/dL in women. It is worth nothing that, in that study, hyperuricemia was observed significantly more frequently in patients with coexisting AF (48% vs. 34%, *p* = 0.01) [32]. Shimuzu et al. also analyzed patients with HFpEF and showed similar dependencies [33]. In their study, hyperuricemia was defined as UA ≥ 7.0 mg/dL. Patients with hyperuricemia had arterial hypertension, diabetes, and both CKD (67%) and AF (48%) significantly more frequently than patients without hyperuricemia. It is worth noting that, in our study, when the median UA concentration was used as the criterion for dividing the population into two subgroups, we did not find any significant differences in the occurrence of comorbidities, apart from reduced EF, between the two groups. Shimizu et al. also studied the prognostic value of hyperuricemia. They showed that hyperuricemia, but not CKD, was an independent predictor of all-cause death (HR: 1.98, 95% CI: 1.04–3.79; *p* = 0.04, in multivariate cox proportional hazard model) [32]. In the present study, we did not evaluate the prognostic significance of hyperuricemia, but the common denominator between studies was that they showed that the UA concentration could be interpreted separately from renal function.

### 4.3. Uric Acid Separate from Potential Drug Effects

In the second part of the analysis, we assessed the extent to which hyperuricemia might be related to the use of top-class drugs for HFrEF pharmacotherapy. A previous study by Shimizu and colleagues showed that ACEi, beta-blockers, and diuretics were used more frequently in the group with hyperuricemia, compared to a group without hyperuricemia [32]. It is well known that both diuretics and beta-blockers are associated with increases in UA levels, and that ACEi and ARB lower UA levels [3,4]. However, beta-blockers, diuretics (loop diuretics and ARA), and ACEi or ARB (which block the renin-angiotensin–aldosterone system) are the basis of HFrEF pharmacotherapy [2]. Therefore, to minimize the impact of standard pharmacological treatment on the obtained results, we used the median UA concentration, instead of the optimal UA cut-off point from the ROC curve, to predict a reduced EF (<40%) in the different treatment subgroups. As a result, in comparing patients in with high and low UA, only patients that used diuretics showed a significant difference in the proportion of patients with EF < 40%. This confirmation of the relationship between the use of diuretics and hyperuricemia was not surprising; however, in our opinion, the persistent correlation between UA and EF among patients that used diuretics indicated that this relationship was independent of the use of this class of drugs. It is worth noting that the correlation between UA and EF was the same for the group of patients taking diuretics as it was for the larger groups analyzed. Moreover, in those groups, we found that neither the UA nor the EF were significantly correlated with the eGFR.

### 4.4. Cause or Effect, Marker, or Risk Factor?

Ultimately, it remains controversial whether high UA alone leads to impaired cardiac function or whether it is merely a marker of HF status. However, one of our results may shed new light on this issue. Although it can be assumed that permanent AF is the form of arrhythmia that most significantly impairs cardiac systolic function, in our population, permanent AF occurred with a similar frequency in the subgroups divided by the median UA concentration. Moreover, permanent AF had no significant effect on whether patients had UA levels above the median. Evidence that emerged in the last year showed that drugs that lowered UA concentrations had positive effects on heart function and structure and reduced the risk of HF symptoms [34,35]. Taking those data into consideration, the results of the present analysis suggested that, in addition to serving as a marker of reduced EF in patients with AF, hyperuricemia should be treated as an independent risk factor for left ventricular dysfunction.

### 4.5. Limitations

Our study had some limitations. First, the registry protocol did not provide tools for performing a prospective follow-up. Second, no information was available on the use of drugs for reducing UA. Moreover, there were no data on the doses of the drugs that we included in the regression analysis or on the specific molecules used (apart from ARA). In addition, the correlations demonstrated in this study, despite their statistical significance, were weak [36,37]. Therefore, significance of the correlation results, regardless of the obtained statistical significance, may be debatable. However, in the authors’ opinion, both further regression analyses and clinical experience justify the final conclusion of the study. Despite these limitations, to our knowledge, this study was the first to show an association between UA and EF in a large cohort of patients with AF, and this association persisted regardless of the patient eGFR or the drugs used.

## 5. Conclusions

This study demonstrated an association between reduced EF and UA levels in patients with AF that had normal renal function. Moreover, we found that this association was independent of eGFR. Higher UA concentrations indicated lower EF values, and the UA concentration cut-off point for predicting an EF < 40% was 6.69 mg/dL. We also showed that the use of drug classes known to affect UA levels, including diuretics, did not affect the relationship between EF and UA in patients with AF and a normal eGFR.

Highlights

1. In patients with AF, hyperuricemia is a marker of reduced EF.
2. In patients with AF and eGFR >60 mL/min/1.73 m^2^, the relationship between UA and EF

(a) Is independent of renal function; and
(b) Is independent of the use of drugs typical for treating heart failure

## Figures and Tables

**Table 1:** General characteristics of study patients with atrial fibrillation (*n* = 829).

Demographics and Comorbidities	Number (%) or Mean (±SD)	
Women	366 (44%)	
Age	72 (±11)	
BMI	29.5 (±5.5)	
Arterial hypertension	175 (86%)	
Diabetes	284 (34%)	
MI	204 (24.6%)	
Previous stroke	107 (13%)	
Vascular disease ^a^	526 (63%)	
Permanent AF	233 (27%)	
EF < 40%	135 (16%)	
**Pharmacotherapy**	**Number (%)**	
BB	726 (87.6%)	
ACEi	519 (62.6%)	
ARB	155 (18.7%)	
ARA	359 (43%)	
Diuretics ^b^	599 (72%)	
**Laboratory Results**	**Mean (median)**	**Standard Deviation (min–max)**
eGFR (mL/min/1.73 m^2^)	74.3	13.75
UA (mg/dL)	6.56 (6.43)	1.77 (0.44–16.55)
TC (mg/dL)	171.8	52.68
LDL (mg/dL)	100.91	44.59
TG (mg/dL)	125.84	60.88
HDL (mg/dL)	49.69	21.45

Abbreviations: BMI, body mass index; MI, myocardial infarction; AF, atrial fibrillation; EF, ejection fraction; BB, beta-blockers; ACEi, angiotensin converting enzyme inhibitors; ARB, angiotensin receptor blockers; ARA, aldosterone receptor antagonists; eGFR, estimated glomerular filtration rate; UA, uric acid; TC, total cholesterol; LDL, low-density lipoproteins; TG, triglycerides; HDL, high-density lipoproteins. ^a^ including at least one of the following vascular diseases: myocardial infarction, coronary arterial disease, peripheral arterial disease, past coronary-aortic bypass graft surgery, percutaneous coronary intervention, or atherosclerotic plaque in the aorta. ^b^ diuretics: any and all diuretics, including ARA or/and loop diuretics.

**Table 2:** Results of the correlation analysis for the entire study population.

Variable	eGFR (mL/min/1.73 m^2^)	UA (mg/dL)	EF (%)	Age (years)	BMI (kg/m^2^)
**eGFR (mL/min/1.73 m^2^)**	1.00	−0.07	−0.05	0.05	**−0.12**
**UA (mg/dL)**	−0.07	1.00	**−0.15**	0.00	−0.04
**EF (%)**	−0.05	**−0.15**	1.00	−0.03	0.02
**Age (years)**	0.05	0.00	−0.03	1.00	**−0.20**
**BMI (kg/m^2^)**	**−0.12**	−0.04	0.02	−0.20	1.00

Values are correlation coefficients (r). Abbreviations: eGFR, estimated glomerular filtration rate; UA, uric acid; EF, ejection fraction; BMI, body mass index. Significant correlations are shown in bold.

**Table 3:** Results of the multiple regression analysis for EF prediction before backward stepwise elimination of insignificant variables.

Variable	Correlation Coefficient (beta)	Regression Coefficient	T	Significance—*p*
**Age (years)**	−0.026	−0.030	−0.622	0.534
**BMI (kg/m^2^)**	0.001	0.002	0.020	0.984
**eGFR (mL/min/1.73 m^2^)**	−0.055	−0.048	−1.314	0.189
**UA (mg/dL)**	**−0.150**	**−1.082**	**−3.597**	**0.000**

Abbreviations: eGFR, estimated glomerular filtration rate; UA, uric acid; EF, ejection fraction; BMI, body mass index. Significant results are shown in bold.

**Table 4:** Comparison of groups, based on whether the UA concentration was above or below the median value in the study population (6.43 mg/dL).

Demographics and Comorbidities	UA < 6.43 mg/dL *n* = 412	UA ≥ 6.43 mg/dL *n* = 417	*p* Value
	Number (%) or Mean (±SD)	Number (%) or Mean (±SD)	
**Women**	172 (42%)	194 (46.5%)	*p* = 0.166
**Age (years)**	72.46 (±10.77)	72.86 (±11.39)	*p* = 0.608
**BMI (kg/m^2^)**	29.89 (±5.34)	29.08 (±5.7)	*p* = 0.055
**Arterial hypertension**	361 (87%)	354 (85%)	*p* = 0.254
**Diabetes**	139 (34%)	145 (35%)	*p* = 0.754
**MI**	100 (24%)	104 (25%)	*p* = 0.823
**Previous stroke**	57 (14%)	50 (12%)	*p* = 0.424
**Vascular disease ^a^**	261 (63%)	265 (64%)	*p* = 0.952
**Permanent AF**	105 (25.5%)	118 (28%)	*p* = 0.361
**EF < 40%**	46 (11%)	89 (21%)	***p*** **< 0.001**
**Pharmacotherapy**	**Number (%)**	**Number (%)**	
**BB**	353 (86%)	373 (89%)	*p* = 0.116
**ACEi**	256 (62%)	263 (63%)	*p* = 0.816
**ARB**	75 (18%)	80 (19%)	*p* = 0.729
**ARA**	160 (39%)	199 (48%)	***p*** **= 0.01**
**Diuretics ^b^**	276 (67%)	323 (77.5%)	***p*** **< 0.001**
**Laboratory Results**	**Mean (±SD)**	**Mean (±SD)**	
**eGFR (mL/min/1.73 m^2^)**	75.5 (±13.9)	73.05 (±13.91)	***p*** **= 0.01**
**UA (mg/dL)**	5.21 (±0.96)	7.89 (±1.34)	***p*** **< 0.001**
**TC (mg/dL)**	168.36 (±51.63)	175.08 (±53.51)	*p* = 0.076
**LDL (mg/dL)**	99.22 (±44.08)	102.53 (±44.08)	*p* = 0.303
**TG (mg/dL)**	114.65 (±56.54)	136.49 (±63.07)	***p*** **< 0.001**
**HDL (mg/dL)**	50.23 (±25.94)	47.23 (±16.0)	*p* = 0.054
**EF (%)**	51.43 (±10.87)	48.53 (±13.79)	***p*** **= 0.002**

Abbreviations: UA, uric acid; BMI, body mass index; MI, myocardial infarction; AF, atrial fibrillation; EF, ejection fraction; BB, beta-blockers; ACEi, angiotensin converting enzyme inhibitors; ARB, angiotensin receptor blockers; ARA, aldosterone receptor antagonists; eGFR, estimated glomerular filtration rate; UA, uric acid; TC, total cholesterol; LDL, low-density lipoproteins; TG, triglycerides; HDL, high-density lipoproteins. ^a^ including at least one of the following vascular diseases: myocardial infarction, coronary arterial disease, peripheral arterial disease, past coronary-aortic bypass graft surgery, percutaneous coronary intervention, or atherosclerotic plaque in the aorta. ^b^ diuretics: any and all diuretics, including ARA or/and loop diuretics. Significant *p*-values are shown in bold.

**Table 5:** Results of correlation analysis for patients that used diuretics.

Variables	eGFR (mL/min/1.73 m^2^)	UA (mg/dL)	EF (%)	Age (years)	BMI (kg/m^2^)
**eGFR (mL/min/1.73 m^2^)**	1.00	−0.05	−0.02	0.06	**−0.13**
**UA (mg/dL)**	−0.05	1.00	**−0.15**	0.02	−0.08
**EF (%)**	−0.02	**−0.15**	1.00	−0.06	0.01
**Age (years)**	0.06	0.02	−0.06	1.00	**−0.17**
**BMI (kg/m^2^)**	**−0.13**	−0.08	0.01	**−0.17**	1.00

Values are correlation coefficients (r). Abbreviations: eGFR, estimated glomerular filtration rate; UA, uric acid; EF, ejection fraction; BMI, body mass index. Significant correlations are shown in bold.

## Data Availability

All relevant data are contained within the article.
